# The relationship between individual characteristics and HIV-related stigma in adults living with HIV: medical monitoring project, Florida, 2015–2016

**DOI:** 10.1186/s12889-020-08891-3

**Published:** 2020-05-19

**Authors:** Renessa Williams, Robert Cook, Babette Brumback, Christa Cook, Miriam Ezenwa, Emma Spencer, Robert Lucero

**Affiliations:** 1 University of Florida College of Nursing, Gainesville, FL USA; 2 Department of Epidemiology, University of Florida College of Public Health and Health Professions & College of Medicine, Gainesville, FL USA; 3 Department of Biostatistics, University of Florida College of Public Health and Health Professions & College of Medicine, Gainesville, FL USA; 4 Department of Nursing Systems, College of Nursing, University of Central Florida, Orlando, FL USA; 5 Department of Biobehavioural Nursing, University of Florida College of Nursing, Gainesville, FL USA; 6 HIV/AIDS Surveillance Program, Florida Department of Health, Tallahassee, FL USA; 7 Center for Latin American Studies, Department of Family, Community and Health Systems Science, University of Florida College of Nursing, Gainesville, FL USA

**Keywords:** HIV, Depression, HIV-related stigma, Negative self-image stigma, Personalized stigma, Anticipated stigma, Medical monitoring project

## Abstract

**Background:**

Human Immunodeficiency Virus (HIV) disproportionately affects the Southern United States, accounting for approximately 46% of people living with HIV. HIV-related stigma is recognized as a barrier to testing, treatment, and prevention efforts. However, little is known about HIV-related stigma experiences in Florida. Using data collected from the Florida Medical Monitoring Project, we sought to examine individual characteristics associated with HIV-related stigma.

**Methods:**

We analyzed secondary data from the 2015–2016 Medical Monitoring Project in Florida (*n* = 603). Stigma was measured using the 10-item HIV Stigma Scale. Exploratory factor analysis of the HIV Stigma Scale revealed three subscales: negative self-image, anticipated, and personalized stigma. Bivariate and multivariate regression models were used to determine the individual characteristics associated with the HIV Stigma Scale.

**Results:**

Multivariate analysis indicated that people with severe depression scores (OR: 3.13; CI: 1.38–7.13) and persons with disability (OR: 1.64; CI: 1.03–2.61) had significantly increased odds of higher overall stigma. In the subscale analyses, negative self-image was significantly associated with alcohol misuse (OR: 2.02; CI: 1.15–3.56) depression (OR: 2.81; CI: 1.38–5.72) and/or those who identify as homosexual (OR: 0.54; CI: 0.31–0.93). Anticipated stigma was significantly associated with people who had mild-moderate depression (OR: 3.03; CI: 1.20–7.65), severe depression (OR: 2.87; CI: 1.38–5.98), identified as Black (OR: 0.60; CI: 0.37–0.98), non-injection drug use (OR: 0.55; CI: 0.33–0.91), and/or people aged 50 years and older (OR: 0.28; CI: 0.09–0.82). Personalized stigma was not associated with any of the variables examined.

**Conclusions:**

The implications of these findings reveal that certain individuals are more vulnerable to stigma. Researchers could consider distinct stigma interventions strategies based on the characteristics of specific individuals (i.e., targeting depression, disability, sexual orientation, avoidant coping, racial/ethnic groups, and youth) in Florida.

## Background

Southern United States (U.S.), including Florida, is disproportionally affected by the Human Immunodeficiency Virus (HIV). This region accounts for approximately 46% of people living with HIV (PLWH) in the U.S. with new diagnoses of HIV occurring amongst men who have sex with men, racial/ethnic minorities, and certain geographic hotspots [1, 2]. In February 2019, the United States Department of Health and Human Services (DHHS) announced a plan to End the HIV Epidemic by reducing nationwide HIV transmission rates by 90% by 2030 [2]. The plan includes four pillars: diagnosing all PLWH, engaging them in rapid antiretroviral treatment to achieve and maintain viral suppression, protecting persons at risk for HIV using biomedical intervention such as pre-exposure prophylaxis (PrEP), and responding to rapidly growing transmission of HIV [3]. However, one of the challenges to achieving the DHHS goal is how to better understand the role HIV-related stigma plays in HIV prevention, testing, treatment, and viral suppression [2].

Stigma is conceptualized as a deeply discrediting attribute that is imposed upon by society [4]. In the context of HIV, stigma is based on the incorrect beliefs and attitudes that devalue PLWH [5]. The PLWH may experience enacted, anticipated, and internalized stigma. PLWH may directly experience enacted stigma, defined as being stereotyped, excluded, or discriminated against due to one’s HIV status [6]. This experience may serve as a barrier to seeking treatment and are theorized to have negative effects on health outcomes such as viral load and CD4 counts [6]. PLWH may also experience anticipated stigma, or the expectation that there will be negative reactions toward their HIV status [6]. Such anticipated stigma could greatly impact medication adherence, as PLWH may actively avoid testing, services, and treatment programs [6]. Lastly, HIV-related stigma may also be internalized, resulting in feelings of negative self-image, shame, and guilt because of one’s HIV status [6]. Possible consequences of internalized stigma include low self-esteem, depression, and helplessness [6].

According to Earnshaw and Chaudoir [7], the assessment of enacted, anticipated, and internalized stigma are essential to predict health outcomes, target potential interventions, and identify HIV-related stigma in affected populations. The 40-item HIV Stigma Scale is one of the only measures that differentiates between these three dimensions of stigma in a single instrument and produces an overall stigma score [6, 7]. A shorter, 10-item version of the scale has been psychometrically evaluated in youth living with HIV, but has yet to be evaluated among adults [8]. Further research is needed to fully understand the association between individual characteristics and HIV-related stigma in adults if characteristics associated with each of the dimensions of HIV-related stigma are evaluated independently.

The HIV-related Stigma, Engagement in Care, and Health Outcomes framework [6] explains that stigma may be exacerbated by aspects of one’s identity (i.e., age, race, gender, disability, or sexual orientation) or may impair health outcomes through other mechanisms (i.e., interpersonal factors, mental health, stress, and psychological resources) [6]. Several studies indicate that the nature of stigma can be population specific [5, 8]. For example, in a cross-sectional surveillance study of a representative sample of PLWH in the U.S., internalized stigma was significantly higher among Hispanics compared to non-Hispanics [9]. However, in a sample of PLWH in New York City, internalized stigma was not significantly associated with race, but there were gender-specific differences of both internalized and anticipated stigma [10]. These mixed findings suggest that stigma cannot be generalized across races and may be experienced differently among different populations. Therefore, it is unknown if the same results would exist among PLWH in Florida. By identifying which individual characteristics are associated with overall and subdimensions of HIV-related stigma, future stigma-reduction efforts could be designed for people who face the greatest burden in Florida. Using data collected from the Medical Monitoring Project (MMP) in Florida, our study (1) identified whether there are different dimensions of HIV-related stigma being measured by the HIV Stigma Scale and (2) examined the association between individual characteristics and HIV-related stigma dimensions in adults living with HIV.

## Methods

We conducted a cross-sectional analysis of interview data collected in the Florida MMP between 2015 and 2016. The MMP, funded by the Centers for Disease Control and Prevention (CDC) [11], is an annual HIV supplemental surveillance project in which PLWH receiving or not receiving HIV-related care are surveyed through structured interviews and medical record abstraction about their clinical and behavioural characteristics across 23 jurisdictions in the U.S. and Puerto Rico. Data were collected through face-to-face and telephone interviews by MMP data collectors at the Florida Department of Health [11]. Eligible participants were people diagnosed with HIV, 18 years of age or older, and residents of Florida as of December 31, 2014 and 2015 [11]. A complete description of the MMP interview data weighting methods and sampling approach is described by the U.S. CDC on their website [11]. We received Institutional Review Board approval from our institution.

### Measures

#### HIV-related stigma

Stigma was assessed using the HIV Stigma Scale modified by Wright et al. [8], in which respondents rated their agreement with ten statements (Table 1). The HIV Stigma Scale assesses “negative self-image,” “personalized stigma,” “disclosure concerns,” and “public attitude concerns” [8]. According to Earnshaw and Chaudoir [7], the negative self-image subscale theoretically represents internalized stigma, or feelings of self-shame, guilt, or blame due to HIV [12]. Personalized stigma is proposed to represent enacted stigma [7], or negative consequences of learning one’s HIV status [12]. Disclosure concerns refers to feeling worried or careful about revealing one’s HIV status [12]. Lastly, public attitude concerns are based on how others view HIV [12]. Disclosure concerns and public attitudes are proposed to represent anticipated stigma [7]. Responses to the 10-item HIV Stigma Scale are measured on a 5-point Likert scale (strongly disagree = 1 somewhat disagree = 2, neutral = 3 somewhat agree = 4, strongly agree = 5). Overall stigma scores ranged from 0 to 100. Furthermore, each subscale had a continuous score in increments of ten, which depended on the number of items for each subscale. For example, if a subscale has three items (i.e., negative self-image, personalized stigma), the score would range from 0 to 30. If a subscale has four items (i.e., anticipated stigma), the score would range from 0 to 40. A categorical measure was created for overall stigma and each subscale based on the median, resulting in a dichotomous measure of higher stigma and lower stigma, as previously done in other investigations [13].

**Table 1:** Exploratory factor analysis with oblimin rotation^a^

Questionnaire Items	Factor 1: Negative Self-Image Stigma	Factor 2: Personalized Stigma	Factor 3: Anticipated Stigma	*h*^2^
I have been hurt by how people reacted to learning I have HIV	0.04	**0.61**	0.04	0.41
I have stopped socializing with some people because of reactions to HIV status	0.04	**0.85**	−0.06	0.72
I have lost friends by telling them I have HIV	−0.07	**0.76**	0.04	0.57
I am very careful about who I tell I have HIV	−0.02	−0.07	**0.44**	0.17
I worry that people who know I have HIV will tell others	0.05	0.11	**0.46**	0.29
I feel I’m not good a person as others because of HIV	**0.74**	0.02	0.03	0.58
Having HIV makes me feel unclean	**0.83**	−0.04	0.01	0.68
Having HIV makes me feel that I’m a bad person	**0.75**	0.02	−0.01	0.57
Most people think that a person with HIV is disgusting	0.14	0.01	**0.52**	0.35
Most people with HIV are rejected when others find out	−0.07	0.07	**0.71**	0.50
Cronbach Alpha	0.81	0.78	0.64	

^a^Loadings ≥ 0.40 are bolded

#### Individual characteristics

Individual characteristics, including race/ethnicity, gender, age, disability, poverty, sexual orientation, education, country of birth, alcohol misuse, depression, anxiety, and non-injection drug use (excluding alcohol), were based on self-report and validated measures of mental health (Table 2). A persons race was categorized as Black, White, or other. The other racial categories including Asian, Native American/Alaskan Native, and Native Hawaiian/Pacific Islander were collapsed into one group. Individuals were identified as having a disability if they responded “yes” to having serious difficulty in any one of the following areas: deaf or serious difficulty hearing; blind or serious difficulty seeing; difficulty concentrating, remembering or making decisions; difficulty with walking or climbing stairs; any difficulty with dressing, bathing or doing errands alone such as visiting a doctor’s office or shopping. Poverty was defined by the DHHS guidelines as “at or above poverty level” or “below poverty level” based on a predetermined graduated income level given the number of individuals in a household in 2014 and 2015 [14]. Sexual orientation was categorized as homosexual, heterosexual, and bisexual/other. Other sexual orientation included persons who answered they were “something else.” Alcohol misuse was calculated as persons who reported heavy drinking patterns (i.e., binge drinking on five or more days in the past month) or binge drinking patterns (i.e., five or more alcoholic drinks for males or four or more alcoholic drinks for females on the same occasion at least 1 day in the past month) [15]. Depression was measured by the eight-item Patient Health Questionnaire, a diagnostic tool to assess the severity of current depression. Depression categories were created based on the Kroenke criteria: no depression (0–4); mild-moderate depression (5–14) ; and severe depression (≥ 15) [16]. The seven-item Generalized Anxiety Disorder Scale was used to assess the severity of anxiety. Score were categorized in three groups also based on the Kroenke criteria: no anxiety (0–4); mild- moderate anxiety (5–14) ; and severe anxiety (≥ 15) [17]. Lastly, non-injection drug use was classified as a binary variable (i.e., yes/no) that referred to those who endorsed having smoked, snorted, inhaled or ingested drugs like marijuana, methamphetamine, cocaine, crack, or certain prescription drugs in the past 12 months [11].

**Table 2:** Individual characteristics among persons living with HIV in 2015 and 2016 (*N* = 603)

Characteristics	N (weighted %)^a^
**Disability Status**	
Persons with disability	295 (48%)
Persons without disability	307 (51%)
**Gender**	
Female	216 (30%)
Male	387 (70%)
**Race**	
White	310 (49%)
Black	272 (47%)
Other^b^	20 (4%)
**Ethnicity**	
Hispanic/Latino	130 (21%)
**Sexual Orientation**	
Homosexual	186 (33%)
Heterosexual	373 (59%)
Bisexual/Other	43(9%)
**Age (years)**	
18–29	38 (8%)
30–49	358 (55%)
50+	207 (37%)
**Education**	
Less than high school	136 (21%)
High school diploma or equivalent	159 (26%)
Greater than High school	308 (53%)
**Poverty**	
Above Poverty Level	275 (50%)
At or below poverty Level	285 (43%)
**Country of Birth**	
Non-foreign Born	468 (79%)
Foreign Born	128 (20%)
**Non-injection Drug User**	
Yes	146 (27%)
No	452 (72%)
**Alcohol Misuse**^c^	
Yes	99 (19%)
No	496 (79%)
**Anxiety**	
No anxiety	465 (77%)
Mild-Moderate Anxiety	91 (15%)
Severe Anxiety	41 (6%)
**Depression**	
No Depression	484 (81%)
Mild-Moderate Depression	32 (5%)
Severe Depression	87 (14%)

^a^Percentages may not add up to 100% due to non-response

^b^Includes Asian, Alaskan Native, Native American, and Pacific Islander

^c^Defined as heavy drinking (binge drinking on 5 or more days in the past month) or binge drinking (i.e., five or more alcoholic drinks for males or four or more alcoholic drinks for females on the same occasion at least one day in the past month)

#### Factor analysis and factor scores

Exploratory factor analysis was performed to determine the underlying dimensions of stigma captured by the HIV Stigma Scale among adult (over 18 years old) PLWH in Florida. Three factors were revealed (Table 1). Three items loaded on the first and second factors and four items loaded on the third factor. All items loaded with a factor loading of ≥ 0.40 and none loaded on multiple factors [18]. The first two factors (i.e., negative self-image and personalized stigma) that we found were consistent with a previous psychometric evaluation using the 10-item HIV Stigma Scale by Wright et al. [8]. Therefore, the content of the factors was interpreted to represent the dimensions of negative-self-image, personalized, and anticipated stigma. Cronbach’s alpha values for the negative self-image, personalized, and anticipated stigma factors were 0.81, 0.78, and 0.64, respectively.

### Statistical analysis

Descriptive analyses were conducted using survey weights assigned by the CDC to summarize individual characteristics of the participants. The associations between overall HIV-related stigma, the underlying subdimensions of stigma, and individual characteristics were evaluated using the modified Rao Scott *Chi-*square test. Bivariate results significant at *p* ≤ 0.25 were included in a multivariate logistic regression model [19]. Odds ratios (OR) and their corresponding 95% confidence intervals (CI) were determined. All statistical analyses were conducted using SAS 9.4 [20].

## Results

### Individual characteristics

Six hundred and three PLWH in Florida participated in the 2015 and 2016 MMP data collection cycles. Table 2 represents the distribution of individual characteristics across the sample (*n* = 603) after accounting for weighting. The majority (70%) of the sample were males and the minority were females (30%). Forty-seven percent were Black, 49% White, and 4% were from other racial/ethnic groups. Twenty-one percent of the sample reported being Hispanic. Persons with a disability comprised 48% of the sample. The mean age was 49 years old (SD ±0.64). More than half of the sample (53%) had an education greater than a high school diploma. Most of the participants were heterosexual (59%) and born in the United States (80%). However, 43% lived at or below the poverty level. Twenty-seven percent reported using non-injection drugs in the past 12 months, and 19% had misused alcohol during the past month, before participating in the MMP. Six percent of the sample had severe anxiety, 15% mild-moderate anxiety, and 77% had no symptoms of anxiety. Fourteen percent of the sample had severe depression, 5% met the criteria for mild- moderate depression, and 81% had no symptoms of depression.

### HIV-related stigma subscales

The median scores for overall HIV-related stigma, negative self-image, personalized, and anticipated stigma are presented in Table 3. Overall stigma had a median of 37.5 (range: 0–100) with 299 participants scoring above the median and 281 participants scoring below. On the negative self-image subscale, the median score was 0 (range 0–30), with 208 participants scoring above the median and 387 participants scoring zero. The personalized stigma subscale had a median of 27.5 (range: 0–30). Of those, 306 participants scored above the median and 286 participants scored below. Finally, the anticipated stigma subscale had a median of 7.5 (range: 0–40), with 324 participants scoring above the median and 271 participants scoring below.

**Table 3:** HIV-related stigma subscale scores among PLWH in Florida (*N* = 603)

HIV- related Stigma Subscale	Median Scores	Higher Stigma^b^	Lower Stigma^c^
Overall^a^ (*n* = 580)	37.5	299 (49%)	281 (51%)
Negative Self-Image (*n* = 592)	0.0	208 (34%)	387 (66%)
Personalized (*n* = 595)	7.5	306 (50%)	286 (50%)
Anticipated (*n* = 595)	27.5	324 (53%)	271 (47%)

^a^Overall stigma and HIV-related stigma subscales did not represent all 603 participants due to missing data

^b^Participants who scored above the median

^c^Participants who scored below the median

### Individual characteristics and HIV-related stigma

Table 4 shows multivariate regression results where PLWH with severe depression (*OR*: 3.13; CI:1.38–7.13) or a disability (*OR:*1.64; CI: 1.03–2.61) had higher odds of experiencing overall stigma. In the subscale analysis, PLWH with alcohol misuse (*OR:* 2.02; CI: 1.15–3.56) or severe depression (*OR:* 2.81; CI: 1.38–5.72) had higher odds and those who reported homosexual orientation (*OR*: 0.54; CI: 0.31–0.93) had lower odds of negative self-image stigma. PLWH with mild-moderate depression (*OR*: 3.03; CI: 1.20–7.65) or severe depression (*OR*: 2.87; CI: 1.38–5.98) had higher odds and those who identified as Black (*OR*: 0.60; CI: 0.37–0.98), were non-injection drug users (*OR*: 0.55; CI: 0.33–0.91), or were 50 years and older (*OR*: 0.28; CI: 0.09–0.82) had lower odds of anticipated stigma. There were no statistically significant associations between personalized stigma and any of the individual characteristics.

**Table 4:** Characteristics of PLWH who endorse experiencing higher HIV-related stigma

Characteristics	Overall Stigma OR (95% CI)	Negative Self-Image Stigma OR (95% CI)	Personalized Stigma OR (95% CI)	Anticipated Stigma OR (95% CI)
**Gender**				
Female	Ref	Ref	Ref	Ref
Male	0.62 (0.36–1.08)	1.00 (0.59–1.70)	0.77 (0.50–1.19)	0.67 (0.39–1.17)
**Race**				
White	Ref	n/s^a^	n/s	Ref
Black	1.17 (0.69–2.00)			**0.60 (0.37–0.98)**
Other	1.00 (0.58–1.69)			0.78 (0.25–2.41)
**Ethnicity**				
Non-Hispanic	Ref	n/s	Ref	n/s
Hispanic/Latino	0.79 (0.43–1.42)		0.69 (0.42–1.14)	
**Disability**				
Persons without disability	Ref	Ref	Ref	Ref
Persons with disability	**1.64 (1.03–2.61)**	1.05 (0.65–1.69)	1.27 (0.83–1.93)	1.27 (0.81–2.00)
**Sexual Orientation**				
Heterosexual	Ref	Ref	n/s	Ref
Homosexual	0.65 (0.38–1.13)	**0.54 (0.31–0.93)**		0.59 (0.34–1.04)
Bisexual/Other	1.00 (0.43–2.33)	1.32 (0.59–2.95)		1.05 (0.45–2.46)
**Age (years)**				
18–29	Ref	Ref	n/s	Ref
30–49	1.00 (0.38–2.56)	1.88 (0.72–4.94)		0.61 (0.21–1.84)
50+	0.48 (0.19–1.22)	1.18 (0.45–3.08)		**0.28 (0.09–0.82)**
**Education**				
Less than High school	Ref	n/s	n/s	Ref
High school diploma	0.63 (0.34 – 1.18)			0.64 (0.34–1.20)
Greater than high school	0.85 (0.46–1.56)			0.74 (0.39–1.38)
**Poverty**				
Above Poverty Level	Ref	Ref	n/s	Ref
At or below poverty Level	1.13 (0.70–1.82)	1.41 (0.88–2.24)		0.80 (0.50–1.30)
**Country of Birth**				
Non-foreign Born	Ref	n/s	Ref	n/s
Foreign Born	1.21 (0.69–2.14)		1.53 (0.93–2.52)	
**Non-Injection Drug Use**				
No	n/s	n/s	n/s	Ref
Yes				**0.55 (0.33–0.91)**
**Alcohol Misuse**				
No	n/s	Ref	n/s	n/s
Yes		2**.02 (1.15–3.56)**		
**Depression**				
No Depression	Ref	Ref	Ref	Ref
Mild-moderate Depression	2.92 (0.91–9.43)	0.94 (0.36–2.42)	0.90 (0.33–2.45)	3**.03 (1.20–7.65)**
Severe Depression	**3.13 (1.38–7.13)**	**2.81 (1.38–5.72)**	1.71 (0.85–3.43)	**2.87 (1.38–5.98)**
**Anxiety**				
No Anxiety	Ref	Ref	Ref	Ref
Mild-moderate Anxiety	0.95 (0.51–1.79)	1.38 (0.73–2.62)	1.02 (0.56–1.85)	0.80 (0.43–1.49)
Severe anxiety	1.13 (0.35–3.66)	0.92 (0.33–2.56)	1.51 (0.58–3.91)	1.07 (0.38–3.00)

^a^n/s- Variables were not included if non-significant in the bivariate analysis

## Discussion

### Principal findings

This is one of the first studies to examine the association between individual characteristics and HIV-related stigma in PLWH in Florida. A notable and unexpected finding from this study was the identification of only three subscales within the HIV Stigma Scale after conducting the exploratory factor analysis. Negative self-image and personalized stigma contained the identical items found in the original HIV Stigma Scale. However, anticipated stigma in this study comprised of the four items that made up two of the subscales (i.e., disclosure concerns and public attitudes) identified by Wright et al. [8]. Another notable finding was that depression was the only individual characteristic of PLWH in Florida that was associated with more than one dimension of stigma. This suggests that PLWH who have symptoms of depression may be most affected by the full spectrum of HIV-related stigma. Additionally, we found that stigma is highest in persons who are disabled or people who misuse alcohol. Other factors significantly associated with at least one stigma type included non-injection drug use, older age, and sexual orientation.

### Exploratory factor analysis

The exploratory factor analysis in this study supports the notion that HIV-related stigma comprises three latent structures for adults living with HIV in Florida. While the Wright et al. [8] HIV Stigma Scale sample included predominantly Black youth (i.e., 16–25) to inform the development of the 10-item scale, the scale has not been psychometrically evaluated in samples of adults living with HIV. Our work suggests that adult PLWH in Florida may report the experience of stigma differently than youth. We retained previously used constructs from the HIV Stigma Scale (i.e., negative self-image and personalized stigma) which is consistent with previous work done by Wright et al. [8]. Although these researchers suggested that “disclosure concerns” and the “public attitudes” stigma dimensions are distinct [8], our analysis suggests that these two dimensions encompass one construct, anticipated stigma. We adopted the use of anticipated stigma based on the work of Earnshaw and Chaudoir [7]. In their literature review of conceptualizing and measuring stigma, Earnshaw and Chaudoir [7] suggest that the HIV Stigma Scale dimensions that represent “disclosure concerns” and “public attitudes” measure expectations of negative treatment, which they label anticipated stigma [7].

### Overall stigma

The experience of overall HIV-related stigma was significantly associated with persons living with disability and depression. There is a small amount of work examining persons with disability and HIV-related stigma [21–23], which identified that stigma may exacerbate or alleviate episodes of disability. This may be because people with disabilities face significant barriers to HIV programming and services, including lack of access to health care services, sexual, or reproductive education, poverty, and challenges to social inclusion compared to those who are not disabled [24]. We still lack an understanding of how pervasive HIV-related stigma is among persons with disabilities. Moreover, respondents who showed symptoms of severe depression also had significantly higher overall stigma than persons who did not report depression. It is plausible that stigmatizing reactions and beliefs associated with HIV could evoke feelings of rejection, guilt, or self- blaming in PLWH [25]. Other studies involving PLWH have also found mental health symptoms, including depression are associated with HIV-related stigma [26, 27]. Overall, more research is needed to understand the effects of mental health, disability, and HIV-related stigma to inform future interventions.

### Negative self-image

We found that negative self-image was associated with severe depression and alcohol misuse. Higher levels of HIV-related stigma and depression have been identified in a recent systematic review by Rueda et al. [28]. HIV-related stigma may be related to psychological well-being via unhealthy coping mechanisms such as avoidance of stressors [6, 29]. Previous work done by Turan et al. [6], postulated that substance abuse, including alcohol is a common method of avoidance coping, as it offers a distraction from distressing thoughts associated with stigma. Avoidant coping may occur when PLWH avoid behaviours that remind them about HIV, such as skipping medical appointments or not adhering to antiretroviral medications to lessen the emotional response [6]. Alcohol misuse may further worsen engagement in HIV care, health outcomes (e.g. viral suppression), and mental health. It is plausible that coping mechanisms mediate the relationship between depression and HIV-related stigma [6]. Prospective studies could clarify the relationships between negative self-image and avoidant coping mechanisms, particularly exploring the potential impact of alcohol misuse and viral suppression.

Our results highlight that people who identified as homosexual had significantly lower odds of experiencing negative self-image stigma compared to those who identified as heterosexual in this study. Higher levels of HIV-related stigma among heterosexuals has been documented in several studies [30–32], which could be rooted in fears of being perceived as sexual minority or homophobia. The HIV discourse has been rooted in homophobia and stereotypes on the basis of sexuality since the 1980s [32]. Our findings may reflect this social phenomenon. For instance, people who identify with the lesbian, gay, bisexual, or transgender community who experience negative-self-image, may get involved in activism or community organizations to cope with negative consequences of being diagnosed with HIV [32]. These shared lived experiences, collective identities, and education on HIV/AIDS creates bonds, promotes community awareness, and activism which could ameliorate stigma and its consequences [32]. However, more work is needed to understand how community involvement influences HIV-related stigma.

### Anticipated stigma

Non-injecting drug users had lower odds of experiencing anticipated stigma compared to non-drug users. Previous studies have found both positive and negative associations between non-injection drug use and HIV-related stigma [8, 10]. These inconsistent findings may be related to the potentiating felt effects of pre-existing stigmas related to one’s identity and/or behaviours. For example, non-injecting drugs users may have greater stigma related to drug use compared to HIV-related stigma [8, 33]. On the other hand, non-injecting drugs users may have also developed the ability to cope with anticipated stigma by using substances to mitigate the negative reactions they may receive from others.

Our study also found that individuals who identified as Black had lower odds of experiencing anticipated stigma compared to those who identified as White. Drawing from the minority stress theory [33], minorities are exposed to excessive stress (i.e., stigma, prejudice, and discrimination), which may have significant implications for their mental health [34]. Therefore, it would be expected that non-Whites would have the highest stigma scores due to having multiple stigmatized identities. Yet, our study and several others do not support this notion [35, 36], suggesting that stigma is higher among Whites compared to non-Whites. Furthermore, the prevalence of HIV is greatest among racial/ethnic minorities groups compared to Whites. Therefore, it is plausible that higher stigma among Whites is associated with feeling like the “minority” of the HIV/AIDS community, which increases alienation and shame [37]. In fact, Lekas et al. [37] suggest that non-White PLWH may feel more stigmatized by the larger society but less stigmatized within their own communities. Although non-White PLWH may face stressors associated with stigma, social inequalities, racism, and HIV/AIDS, the minority stress theory does not take into account that positive group identity may be a protective factor against anticipated stigma [33]. Future work could explore the mediating roles of self-protective mechanisms associated with HIV-related stigma. Moreover, exploring racial/ethnic differences of HIV-related stigma in other geographic locations may elucidate these findings.

Anticipated stigma was also significantly lower in adults 50 years and older compared to those younger than 30. Several studies have found a relationship between older age and lower HIV-related stigma [9, 10, 36]. Emlet and colleagues [31] hypothesize that older adults exert more influence on age-related concerns (e.g., fears of aging or getting sick, career transition, familial stress) and are less affected by anticipated stigma, whereas younger individuals may have less knowledge about HIV transmission and risk factors [9, 10, 38]. In the context of PLWH in Florida, individuals between the ages of 20–29 had the highest proportion of new HIV diagnosis as of 2017 [39]. In fact, PLWH younger than 24 years old were the least likely of any age group to be linked to care or achieve viral suppression [39]. Anticipated stigma may prevent younger PLWH from seeking testing and not disclosing their status due to the fear of negative reactions [6]. These results suggest that interventions addressing anticipated stigma tailored towards younger PLWH may be more beneficial, specifically addressing HIV transmission and risk factors.

Participants who were mild-moderately depressed and severely depressed had significantly higher anticipated stigma compared to those with no symptoms of depression. Our results suggest that persons who have depressive symptoms are likely to expect negative reactions from others (e.g., HIV is disgusting, PLWH are rejected when others find out) and endorse these negative stereotypes associated with HIV. Further, Ending the HIV Epidemic includes reducing new infections by encouraging PLWH to take antiretroviral medications as directed to achieve viral suppression, remain undetectable, and eliminate the risk of transmitting HIV (i.e., undetectable = untransmissible) [3]. The public health implications of our findings suggest that these messages could further contribute to anticipated and negative self-image stigma among persons with depression who may find it more difficult to adhere to medication and achieve viral suppression due to the impact of cognitive functioning [40].

### Personalized stigma

A study similar to ours by Algarin et al., revealed that enacted, or personalized stigma, is a prevalent issue in Florida, especially among women and non-white Latinos [41]. A limitation of this study is that the investigators only assessed enacted stigma [41]. Yet, it has been established that PLWH may experience different dimensions of stigma (i.e., negative self-image, personalized, and anticipated) [42]. Nonetheless, we did not find a single individual characteristic that was associated with personalized stigma.

### Limitations

There were several limitations worth mentioning in this study. First, causal relationships between individual characteristics and HIV-related stigma could not be determined due to the cross-sectional nature of the study design. Next, all study measures were self-reported. Self-reported data may underestimate the true experience of behavioural (e.g., non-injection drug use) and sociological phenomena (e.g., HIV-related stigma) due to social desirability bias. Further, we found the anticipated stigma dimension had a lower bound alpha of 0.64 [43]. This may suggest that the 10-item HIV Stigma Scale may not best capture stigma in adults. Additionally, because individuals self-reported their depressive symptoms, our measure of depression may be an overestimation or underestimation of some objective measure of depression [44]. Finally, the HIV Stigma Scale items do not specify when the stigma occurred. This may result in the stigma scale being less sensitive to changes in stigma over time, and thus raises temporality concerns. Despite these limitations, strengths of our study include the use of the MMP. The MMP revised methods in 2015 to expand to all PLWH, including persons engaged in care and persons not receiving HIV care [11]. This has increased the capacity to monitor and understand the needs of PLWH who are less apt to be included in population samples.

## Conclusions

In summary, our findings provide evidence that the HIV Stigma Scale appears to contain three factors for adult PLWH, namely, negative self-image, personalized, and anticipated stigma. Overall stigma, negative self-image, and anticipated stigma were significantly associated with depression, while other individual characteristics were uniquely associated with only one of each of the three dimensions of HIV-related stigma. It may be concluded that exploring negative self-image, personalized, and anticipated stigma in different geographic areas will provide valuable information about these different regions’ distinct influences on the experience HIV-related stigma. Specifically, researchers could consider different stigma interventions strategies based on the needs of specific individuals (i.e., addressing depression, persons with disabilities, sexual orientation, avoidant coping, racial/ethnic groups, and youth) in Florida.

## Data Availability

The data that support the findings of this study are available from the Florida Department of Health, but restrictions apply to the availability of these data, which were used under license for the current study, and so are not publicly available. Data are however available from the authors upon reasonable request and with permission of the Florida Department of Health.

## Abbreviations

HIV: Human Immunodeficiency Virus
PLWH: People living with HIV
PrEP: Pre-exposure prophylaxis
DHHS: Department of Health and Human Services
MMP: Medical Monitoring Project
CDC: Centers for Disease Control and Prevention
OR: Odds Ratio
CI: Confidence Intervals
